# Response of Human Macrophages to Clinically Applied Wound Dressings Loaded With Silver

**DOI:** 10.3389/fbioe.2020.00124

**Published:** 2020-02-25

**Authors:** Patrícia Varela, Lennart Marlinghaus, Susanna Sartori, Richard Viebahn, Jochen Salber, Gianluca Ciardelli

**Affiliations:** ^1^Department of Mechanical and Aerospace Engineering, Politecnico di Torino, Turin, Italy; ^2^Department of Experimental Surgery, Universitätsklinikum Knappschaftskrankenhaus Bochum, Ruhr-University Bochum, Bochum, Germany; ^3^Department of Medical Microbiology, Ruhr-University Bochum, Bochum, Germany

**Keywords:** wound infections, wound dressings, silver, immunomodulation, macrophages

## Abstract

Wound infections constitute an increasing clinical problem worldwide. To reverse this trend, several wound dressings with antimicrobial properties have been developed. Considering the increasing presence of antibiotic-resistant microorganisms, product developers have been focusing their efforts in introducing antibiotic-free antibacterial wound dressings to the market, with silver being the most commonly incorporated antimicrobial agent. In this scenario, gaining information about the microbial and eukaryotic cells’ response to these dressings is needed for a proper selection of antimicrobial dressings for the different cases of infected wounds. In particular, one insufficiently explored parameter is the effect of the dressings on the immunomodulation of macrophages, the main immune cell population participating in the repair process, because of their pivotal role in the transition of the inflammation to the proliferation phase of wound healing. In this work, three different clinically applied antimicrobial, silver impregnated wound dressings were selected: Atrauman^®^ Ag, Biatain^®^ Alginate Ag and PolyMem WIC Silver^®^ Non-adhesive. Antimicrobial susceptibility tests (disk diffusion and broth dilution), cell viability evaluation (CellTiter-Blue^®^) and experiments to determine macrophage polarization (e.g., flow cytometry, ELISA and glucose uptake) were performed after 24 h of incubation. Among all products tested, Biatain^®^ Alginate Ag induced the most evident bactericidal effect on Gram-positive and Gram-negative bacteria, followed by PolyMem WIC Silver^®^ Non-adhesive, but did not show good cytocompatibility *in vitro*. On the other hand, Atrauman^®^ Ag showed excellent cytocompatibility on L929 fibroblasts, HaCaT keratinocytes and THP-1 derived macrophages, but no significant antimicrobial activity was observed. Overall, it was confirmed that macrophages initiate, in fact, an alteration of their metabolism and phenotype in response to wound dressings of different composition in a short period of contact (24 h). M0 resting state macrophages common response to all silver-containing dressings used in this study was to increase the production of the anti-inflammatory cytokine TGF-β, which indicates an acquisition of M2-like macrophages characteristics.

## Introduction

Clinicians are daily faced with the extremely difficult challenge of selecting the most appropriate dressing among those available in the market (Dhivya et al., 2015). The selection must be done for each specific case, by doing a comprehensive assessment of the wound status and collecting clinical data of the patient’s physical features (Baranoski and Ayello, 2012). The market has been introducing dressings with antimicrobial properties in accordance with the prevalent high number of skin and soft tissue infection cases (also known as acute bacterial skin structure infections) of inpatient and outpatient health care (Burnham et al., 2016; Simões et al., 2018). In particular, antibiotic-free antibacterial wound dressings are on the rise, in accordance with the constantly increasing number of bacterial species isolated from non-healing wounds that are resistant to commonly used antibiotics in the hospital setting (Piddock, 2017). Among the available products, silver-based wound dressings definitely dominate the market, due to silver’s broad antimicrobial activity against more than 600 clinically isolated strains of non- and more importantly drug-resistant bacteria (Nam et al., 2015; Zewde et al., 2016; Simões et al., 2018). Generally, the antimicrobial principle of these silver dressings relies on the release of silver ions (Ag^+^) that induce the antimicrobial activity. Silver ions are antibacterial through various mechanisms of action, such as interacting with the bacterial membrane leading to the destabilization of the phospholipidic layer or provoking a decrease of adenosine triphosphate (ATP) levels. Silver ions are also known for causing the induction of an increased production of reactive oxygen species (ROS) and interaction with cytosolic components as enzymes and nucleic acids (Sütterlin et al., 2012). However, these dressings are not sufficiently investigated, especially concerning the effects on immunomodulation of macrophages (Krzyszczyk et al., 2018). Acquisition of data on macrophage-biomaterial interactions is decisive to gain a more complete assessment of the characteristics of each wound product and could lead to the selection of the most appropriate material to prevent or reduce local infection. Wound healing is a highly complex, finely regulated process. Based on our current knowledge, the optimal wound healing stages in chronological order go through haemostasis, inflammation, proliferation and remodeling phases (Velnar et al., 2009; Krzyszczyk et al., 2018). During this process, macrophages are deeply involved, by shifting from a predominant M1 pro-inflammatory population to an anti-inflammatory/tissue-remodeling M2 phenotype, while some populations might share characteristics of both M1 and M2 macrophages (Ferrante and Leibovich, 2012). A very important function of macrophages is their role in the transition from the inflammatory to the proliferation phase in wound healing (Landén et al., 2016). After an injury, during the early stages of repair in cutaneous healing, M1 macrophages are more frequently found (Sindrilaru and Scharffetter-Kochanek, 2013). They are specialized in phagocytosing dead cells and their components, besides attracting other macrophages to the damage location (Hesketh et al., 2017). M1 macrophages are known for being antigen presenting cells and for producing the antibacterial oxidative metabolite nitric oxide (NO). Their metabolism is activated toward the production of high levels of pro-inflammatory cytokines such as IFN-γ, IL-1, IL-6, and TNF-α (Schairer et al., 2012; Novak and Koh, 2013). Typically, M1 macrophages present augmented levels of major histocompatibility complex (MHC) cell surface receptors, known as HLA-DR, and the chemokine receptors CD197 that bind CCL19 and CCL21 (Yamane and Leung, 2016).

In contrast, M2-like macrophages appear in higher quantity during proliferation and remodeling phases of the wound healing process. Different from M1 macrophages, M2-like macrophages express high levels of galactose receptors, mannose receptors (CD206) and hemoglobin scavenger receptors (CD163) (Ferrante and Leibovich, 2012; Novak and Koh, 2013). Moreover, these cells constantly produce anti-inflammatory cytokines such as IL-4 and IL-10, and the growth factor TGF-β that has the important function of inducing proliferation of fibroblasts, leading to remodeling of new extracellular matrix for an optimal wound closure (Berg et al., 2003; Ogle et al., 2016).

Unfortunately, in infected wounds a transition from inflammatory to proliferation/remodeling phases is not achieved and a persistent inflammation environment is evident (Hesketh et al., 2017). In such cases, it is needed to avoid external factors, such as biomaterials composition which would prompt this unfavorable environment. Hence it is of paramount importance to evaluate the immunomodulatory effect that different types of commercially available products induce.

Therefore, this work explores the antimicrobial efficacy of silver containing biomaterials that are clinically used on colonized or infected wounds, with a major focus on the response of macrophages to different wound products. A comparative test of the matrix without and with the antimicrobial agent (silver) is also included, in order to obtain information about different cytocompatibility and macrophage immunomodulation responses to the presence of silver.

## Materials and Methods

### Wound Dressing Materials

The commercially available wound dressings for this research work were selected according to the most applied products in clinical set-ups at the University Medical Centre (UMC) of Bochum in Germany. The selected dressings have the same active component – silver ions – being released in different concentrations/time and from diverse systems: metallic silver, patented ion silver complex salt (patent EP1654013B1, registered by Coloplast) or nanocrystalline silver particles. The characteristics of the chosen untreated wound dressings and their correspondent dressings incorporated with a specific type of silver are described in Table 1. Before starting the biological experiments, disks were cut out of the wound dressings in a non-sterile environment and afterward were sterilized under ultra-violet light for 20 min at a wavelength of 253.7 nm, since it is a simple and non-expensive technique extensively used in the area of medical research, and has a strong antimicrobial activity (Valente et al., 2016). All samples were maintained in sealed sterile petri dishes at 4°C for a maximum of one-week before further use in biological experiments.

**TABLE 1:** Selected commercially available wound dressing products (adapted from the informative documents provided by the suppliers).

Name of product	Base constitution	Quantity and type of silver	Other characteristics
Atrauman^®^	Polyester Mesh	Not present	Porous structure (1 mm pore size) and impregnated with neutral ointment that contains fatty acids to promote wound closure
Atrauman^®^ Ag	Polyamide Mesh	300–500 μg/cm^2^ of unreactive metallic silver Ag(0) that in aqueous or moist environment is rapidly ionized to silver ions Ag^+^	Impregnated with ointment made of ester mixture of natural and vegetable fatty acids (Macrogol 2000)

Biatain^®^ Alginate	Dressing consisting of 85% alginate and 15% carboxymethyl Cellulose	Not present	High dressing integrity and calcium ions are released from the dressing inducing a haemostatic effect
Biatain^®^ Alginate Ag		950 μg/cm^2^ of inorganic salt that releases ionic silver Ag^+^ in the presence of wound exudate	Highly absorbent, and provides a haemostatic effect

PolyMem^®^ WIC Non-adhesive	Polyurethane foam	Not present	
PolyMem WIC Silver^®^ Non-adhesive		124 μg/cm^2^ of nanocrystalline silver particles that release clusters of extremely small and highly reactive silver particles, and silver ions, in wound fluid taken up by the dressing	Contains a humectant (glycerol) that avoids the dressing to dry and to adhere to the wound bed, a non-ionic surfactant (poloxamer 188) that facilitates wound cleansing, and a starch copolymer to enhance the fluid handling properties of the foam

### Culture Conditions of Bacterial and Eukaryotic Cell Lines

Four bacterial strains known to infect open wounds were selected for this work. All strains were obtained from the American Type Culture Collection (ATCC). Two of them are Gram-positive – *Staphylococcus aureus subsp. aureus* Rosenbach (ATCC^®^ 29213^TM^) and *Staphylococcus epidermidis* (Winslow and Winslow) Evans (ATCC^®^ 12228^TM^) – and the others are Gram-negative bacteria – *Escherichia coli* (Migula) Castellani and Chalmers (ATCC^®^ 25922^TM^) and *Pseudomonas aeruginosa* (Schroeter) Migula (ATCC^®^ 27853^TM^). For all experiments, bacterial cells were grown at 37°C for 18h on blood agar plates (mixture of nutrient agar with 5% sheep blood pH ∼7.4).

Fibroblasts, keratinocytes and monocyte-derived macrophages, which are cell populations that constitute skin, were used to evaluate the effects of the dressings on 2D cell culture layers of mammalian cells. For all cell lines, the cell number was determined on a Neubauer counting chamber in the presence of 0.4% trypan blue (Gibco) to exclude non-viable cells.

Murine fibroblasts L929 cell line (German Collection of Microorganisms and Cell Cultures – DSMZ) was cultured in RPMI 1640 medium with stable glutamine (PAN Biotech), supplemented with 10% heat-inactivated Fetal bovine serum - FBS (PAN Biotech), and 100 U/mL penicillin - 0.1 mg/mL streptomycin (PAN Biotech). Cells were maintained in T75 vented cap culture flasks (ThermoFisher Scientific) at 37°C in an atmosphere containing 5% CO_2_, by sub-passage with 0.25% Trypsin 1 mM EDTA (PAN Biotech) at each 2–3 days.

The keratinocyte immortalized cell line HaCaT (Deutsches Krebsforschungszentrum - DKFZ, Heidelberg) was expanded in 4.5 g/L glucose Dulbecco’s Modified Eagle Medium – high glucose DMEM (ThermoFisher Scientific) supplemented with 10% heat-inactivated Fetal bovine serum (FBS) (PAN Biotech), and 100 U/mL penicillin - 0.1 mg/mL streptomycin (PAN Biotech) in T75 vented cap culture flasks (ThermoFisher Scientific) at 37°C and 5% CO_2._ The detachment of adhered cells was performed with TrypLE Express (ThermoFisher Scientific). The splitting ratio was from 1:5 to 1:10.

Human monocytic cell line THP-1 (ATCC) was grown at 37°C and 5% CO_2_ in vertically positioned T75 vented cap culture flasks (ThermoFisher Scientific) in the exact same complete RPMI 1640 medium as previously described for L929 cells. Additional medium was added or renewed every 2–3 days when the maximum cell concentration was reached – 8 × 10^5^ cells/mL.

To induce THP-1 monocyte differentiation to M0 macrophages, monocytes were first seeded on cell culture treated 24-well plates (density of 10^6^ cells/mL) in the presence of 200 ng/mL of phorbol 12-myristate-13-acetate (PMA) (Sigma Aldrich) for 24 h (37°C, 5% CO_2_). Afterward the medium was replaced to complete RPMI and cells were again incubated for 48 h before any further experiment.

### Antibacterial Susceptibility Tests

#### Disk Diffusion

The European Committee on Antimicrobial Susceptibility Testing (EUCAST) guidelines were followed for this experiment (Matuschek et al., 2014). Briefly, well-isolated colonies were selected and immersed in 2 mL of 0.9% NaCl. Density was adjusted to MacFarland 0.5 turbidity standard (corresponds to ∼1-2 × 10^8^ colony forming units per milliliter - CFU/mL). With a sterile swab the inoculum was spread evenly on Mueller-Hinton agar plates.

Afterward, the disks obtained from the antimicrobial dressing materials with 1 cm diameter size were carefully placed on the agar plates. As a first control, disks of untreated materials were placed on the agar to observe if there is not a bacterial growth inhibition without the antibacterial agent. A second control was included in the tests with specific antibiotics known to inhibit the strains growth in order to validate the assay: 1 μg oxacillin for *S. aureus* and *S. epidermidis*, and 300 μg streptomycin for *E. coli* and *P. aeruginosa*. Plates were incubated at 37°C for 16–20 h (in duplicates). Photographs were acquired with a Panasonic Lumix DMC-FZ100 digital camera.

#### Colonies Count to Determine Bacterial Reduction in Broth Medium

In order to determine the bacterial reduction in the presence of the material, the broth microdilution method was performed following the EUCAST recommendation to use the ISO 20776-1:2006 standard, with some modifications. The dressing material was cut to circles of 6.5 mm diameter that were placed in duplicates on the bottom of 96-well plates. Colonies selected from an overnight culture grown on blood agar plates were inserted in a tube with 2 mL 0.9% saline solution NaCl. Turbidity was adjusted to MacFarland 0.5 and the inoculum was diluted 400-fold in Mueller-Hinton broth (∼3.75 × 10^5^ CFU/ml). 100 μL of the bacteria suspension was added on the material on each well. The plate was incubated for 24 h at 37°C. After this period, the turbidity of the well was visualized to determine if the material had sufficient concentration of antibacterial agent to inhibit the growth (minimal inhibitory concentration – MIC) of each bacteria strain. Following to the broth MIC test, the entire volume of the well was spread evenly on blood agar plates. These plates were incubated from 16 to 20 h at 37°C and the colony forming units (CFU) were counted and compared to the initial CFU/mL.

### Cytocompatibility Test on Different Cell Populations of Skin

In a sterile flat bottom 24-well plate, 5 × 10^4^ cells per well for L929 fibroblasts and 5 × 10^5^ cells per well for HaCaT keratinocytes were cultured and incubated at 37°C and 5% CO_2_ for 24 h to allow cell adherence. In the case of THP-1 monocytes, the period of incubation was longer due to the induced stimulation for its differentiation into M0 macrophages as previously described in the section *Culture conditions of bacterial and eukaryotic cell lines*. After this time, the medium of the wells was renewed, and the sterilized materials (6.5 mm diameter) were quickly soaked in cell culture medium and added in triplicates on top of the cells layer on each well. The plate was kept in a humidified incubator (37°C and 5% CO_2_) for 24 h. Afterward, CellTiter-Blue^®^ viability assay was performed following the provider instructions. Briefly, the CellTiter-Blue^®^ is based on the conversion of resazurin (a redox dye) to resorufin (a fluorescent end-product) by living cells. After 2 h of incubation with CellTiter-Blue reagent, the fluorescence intensity was quantified on a black 96 well-plate by measuring the fluorescence at 590 nm, after excitation at 560 nm in a Tecan microplate reader Infinite^®^ 200 PRO. As positive control a lysis solution of 9% Triton^®^ X-100 (Promega) was added to the cells, and as negative control, cells were plated on the Tissue Culture Polystyrene (TCPS). This procedure was adapted from the ISO norm 10993-5:2009 (International Organization for Standardization, 2009).

### Evaluation of the Effects of the Materials on Macrophages

#### Morphological Evaluation

Bright field images were acquired on an Olympus IX51 microscope with a magnification of 20× to observe the morphological characteristics of monocyte-derived macrophages after being exposed to the material for 24 h.

#### Flow Cytometry Analysis

Analysis of the phenotypical characteristics of macrophages in the presence of wound dressings for 24 h was performed by flow cytometry (Partec, Münster, Germany).

For direct labeling of cells, all fluorescein-isothiocyanate (FITC)- or phycoerythrin (PE)- conjugated anti-human monoclonal antibodies (Biolegend UK Ltd) were diluted 1:25 and incubated for 30 min at 4°C. The following antibodies were added to cells suspended in 1.5% paraformaldehyde (PFA): FITC-labeled mouse anti-human CD197 (clone G043H7), PE-labeled mouse anti-human HLA-DR (clone L243), PE-labeled mouse anti-human CD163 (clone GHI/61) and FITC-labeled mouse anti-human CD206 (clone 15-2). As autofluorescence control, unstained M0 macrophages were used. Data was analyzed with FloMax software version 2.3 (Partec).

#### Quantification of Cytokine Production (ELISA)

Legend Max^TM^ ELISA kits with pre-coated plates (BioLegend UK Ltd) were used to measure the pro-inflammatory cytokine IL-1β and anti-inflammatory cytokines, IL-10 and TGF-β, present in the cell culture supernatant of the different conditions tested following the manufacturer’s procedure.

#### Nitric Oxide Detection

Nitrate/Nitrite Colometric assay kit (Cayman Chemical, Biomol GmbH, Germany) was used to quantify the concentration of total NO products (nitrate NO_3_^–^ + nitrite NO_2_^–^) in the supernatant that was produced by macrophages in the presence of six different materials for 24 h. The method was according to manufacturer’s instructions.

#### Glucose Uptake

After 24 h in the presence of the unloaded and antibacterial loaded dressing materials, the glucose uptake by macrophages was evaluated. After washing the wells of the 24-well plates with PBS, RPMI 1640 medium without glucose (Gibco^TM^) supplemented with 100 μM of fluorescent D-glucose analog 2-[*N*-(7-nitrobenz-2-oxa-1,3-diazol-4-yl) amino]-2-deoxy-D-glucose (2-NBDG) (Cayman Chemical, Biomol GmbH, Germany) was added. The plates were incubated at 37°C for 60 min in the dark. The supernatant was removed and the wells were washed once with PBS. Accutase (PAN Biotech) was added to induce the detachment of the cells and the content of the wells was transferred to a black 96-well plate to read fluorescence (excitation 465/emission 540 nm) in a Tecan microplate reader Infinite^®^ 200 PRO.

### Statistical Analysis

All data was first expressed as mean ± standard deviation of three independent assays in triplicate. The statistical analysis was performed with the GraphPad Prism 5.00.288 software by one-way analysis of variance (ANOVA) followed by Bonferroni’s multiple comparison test. Statistically significant values are represented as ^∗^*p* < 0.05, ^∗∗^*p* < 0.01, and ^∗∗∗^*p* < 0.001.

## Results

### Antibacterial Susceptibility to the Clinically Used Antimicrobial Wound Dressings

**FIGURE 1:**
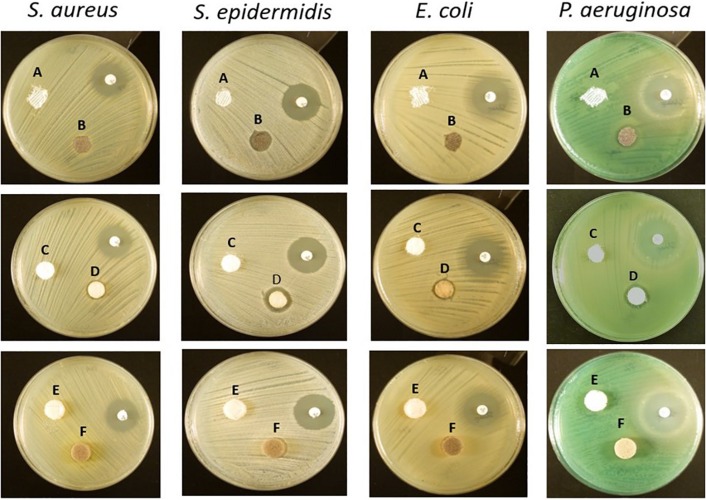
Representative images of the antibacterial susceptibility to the wound dressings by the disk diffusion method. A – Atrauman; B – Atrauman Ag; C – Biatain Alginate; D – Biatain Alginate Ag; E – PolyMem WIC; F – PolyMem WIC Silver. The used non-labeled antibiotic disks on the images were oxacillin (on *S. aureus* and *S. epidermidis* plates) and streptomycin (on *E. coli* and *P. aeruginosa* petri dishes).

The antibacterial efficacy of the selected wound dressings was evaluated on four bacterial strains typically found in wound infections (*S. aureus*, *S. epidermidis*, *E. coli*, and *P. aeruginosa*) (Gjødsbøl et al., 2006; Ibberson et al., 2017). Disk diffusion was first performed to assess the antibacterial capacity of the materials in a warmed and humidified condition. No bacterial inhibition was observed with the untreated materials (Figures 1A,C,E). In our evaluation, it was observed that only Biatain Alginate Ag was able to inhibit the growth of the four strains tested (Figure 1 and Table 2). Atrauman Ag and PolyMem WIC Silver induced an inhibition zone against *S. epidermidis* only (Figure 1 and Table 2).

**TABLE 2:** Diameter of the Halo of bacterial growth inhibition induced by the placed antibacterial commercial wound dressings (*n* = 3).

Dressing material	Diameter of the inhibition zone (mm)			
	
	*S. aureus*	*S. epidermidis*	*E. coli*	*P. aeruginosa*
Atrauman^®^ Ag	–	13.5 ± 0.5	–	–
Biatain^®^ Alginate Ag	12.5 ± 0.5	16.5 ± 0.5	13.0 ± 0	15.5 ± 0.5
PolyMem WIC Silver^®^	–	12.5 ± 0.5	–	–

*Average ± Standard deviation.*

**FIGURE 2:**
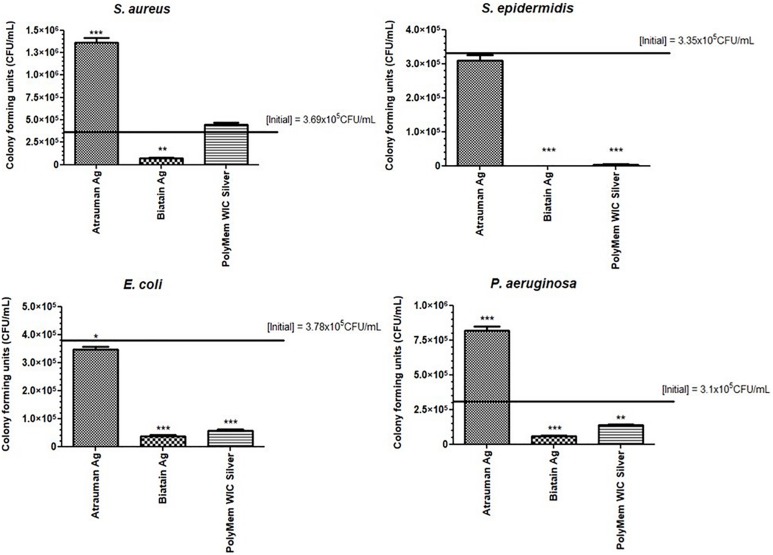
Bacterial growth or reduction (in reference to the initial inoculum) in the presence of the antibacterial materials for 24 h assessed by counting the colony forming units/mL. *p* values were determined for all groups versus initial CFU/mL. Statistical significance was performed with data from three independent experiments and is presented as following *p ≤ 0.05, **p ≤ 0.01, and ***p ≤ 0.001. Error bars represent standard deviation.

Another antibacterial method was performed as complement to the disk diffusion results. In this method, the dressings were completely submersed in Mueller-Hinton broth providing a more liquid environment than disk diffusion and differences on the antibacterial properties were clearly observed. Moreover, with the colony forming units (CFU) counted after 24 h, it was possible to determine the exact reduction of CFU/mL provoked by each antimicrobial dressing. Atrauman Ag seems to prevent the continuous growth of *S. epidermidis* and *E. coli* (Figure 2). In case of *E. coli* this effect was not observed on the disk diffusion test (Figure 1). Biatain Alginate Ag was able to induce 80 to 99% reduction of CFU/mL on all strains tested, showing significantly different values of CFU in the presence of this material versus the initial bacterial concentration (Figure 2). This result in combination with the disk diffusion suggests that Biatain Alginate Ag allows a more rapid dissociation of the inorganic salt in contact with a moist or aqueous environment leading to the release of Ag^+^ in the first 24 h than the other dressings. PolyMem WIC Silver showed efficient antibacterial properties in a liquid medium, in comparison with the disk agar diffusion method. Here a significant reduction of bacterial CFU against *S. epidermidis* and both Gram-negative strains was determined. For *S. aureus* this dressing seems to induce a bacteriostatic effect (Figure 2).

### Bioevaluation of the Cytocompatibility of the Wound Dressings

Three cell populations that are involved in wound regeneration (fibroblasts, keratinocytes and monocyte-derived macrophages) were selected to perform a preliminary direct contact assessment of the biomaterial compatibility *in vitro* on 2D-cell cultures (Shaw and Martin, 2009).

**FIGURE 3:**
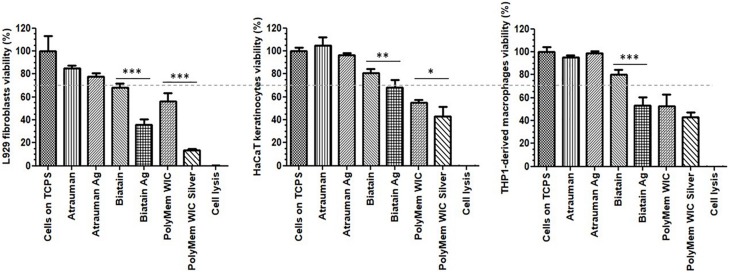
Cell viability in the presence of unloaded and silver loaded dressing materials for 24 h. The dashed line represents 70% viability. The negative control are the cells seeded directly on tissue culture polystyrene (Cells on TCPS) and the positive control are dead cells that were lysed with 9% Triton^®^ X-100 at the endpoint of the assay (Cell lysis). Error bars represent standard deviation. *p* statistical significance values (^∗^*p* ≤ 0.05, ^∗∗^*p* ≤ 0.01, and ^∗∗∗^*p* ≤ 0.001) were determined for unloaded vs. respective silver containing material (*n* = 3).

Among the 6 types of materials, Atrauman and Atrauman Ag showed an excellent cytocompatibility to the three different cell lines (Figure 3). Biatain Alginate Ag leads to the release of the active component’s (Ag^+^), that turned out to be cytotoxic to the populations tested (Figure 3). The lower cell viability can be ascribed to the incorporated Ag^+^ ions due to the statistical difference observed in comparison with silver-free Biatain Alginate, which showed an optimal cell viability (≥70%). Moreover, PolyMem WIC induced a reduction of cell viability by more than 30%. In combination with nanocrystalline silver, a higher reduction of cell viability was observed, showing significant values of ^∗∗∗^*p* ≤ 0.001 for L929 fibroblasts and ^∗^*p* ≤ 0.05 for HaCaT keratinocytes (Figure 3).

### Response of Monocyte-Derived Macrophages to Wound Dressings: Phenotypical and Metabolic Characterization of Macrophages After Being Exposed to the Materials for One Day Period of Contact

After incubating M0 macrophages with the 6 different wound dressings, tests were conducted in order to gain insight in the metabolic mechanisms, that lead to the activation of macrophages in the first 24 h. Therefore, the phenotypical and metabolic profile of the monocyte-derived macrophages were analyzed after an incubation with the material on top of the cell layer for 24 h. For phenotypical characterization, besides obtaining bright field images, macrophages were stained with cell surface markers CD197/HLA-DR and CD163/CD206 that are typically expressed by M1 and M2, respectively, and were analyzed by flow cytometry. For further characterization, metabolic changes were analyzed in terms of cytokine production (IL-1β, IL-10, and TGF- β), nitric oxide (NO) release and glucose uptake.

**FIGURE 4:**
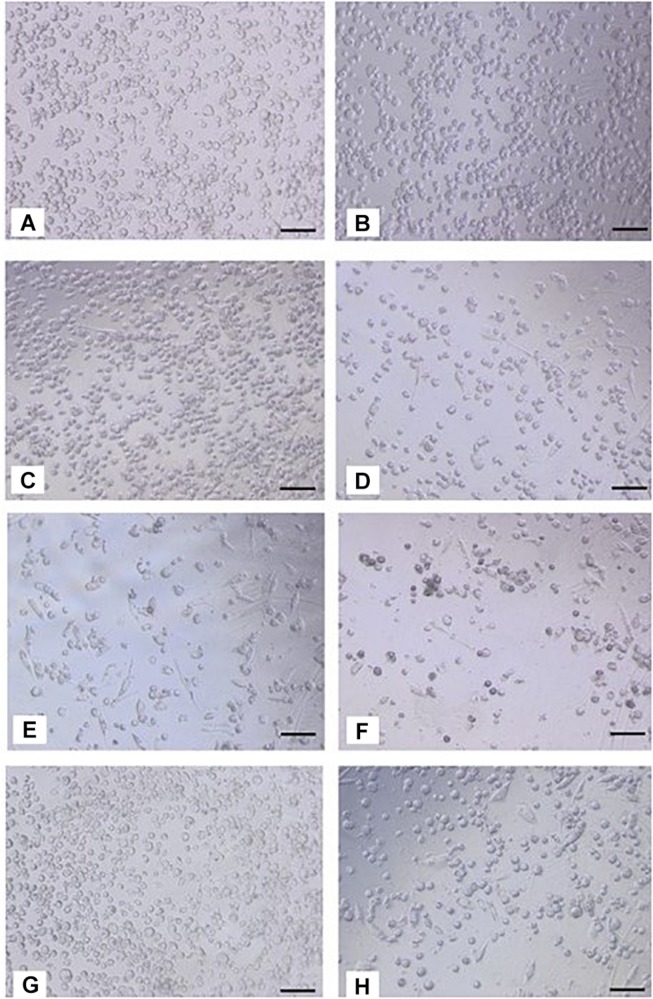
Bright field images of macrophages after 24 h of exposure to silver-free and silver-containing wound dressings. **(A,B)** M0 macrophages on TCPS; **(C)** Atrauman; **(D)** Atrauman Ag; **(E)**, Biatain Alginate; **(F)** Biatain Alginate Ag; **(G)** PolyMem WIC; **(H)** PolyMem WIC Silver. Bar represents 100 μm.

In the presence of silver-free Atrauman, cell morphology was very similar to the M0 macrophages (Figures 4A–C). However, after exposure to Atrauman Ag, the cell morphology was altered in comparison with the control condition (Figures 4A,B,D). The bright field microscopic images show generally a more elongated cell morphology in comparison with the M0 macrophages for the Biatain Alginate dressings (Figures 4A,B,E,F). In the presence of PolyMem alone two different shapes of cell morphology are observed in Figure 4G that shows more rounded cells than stretched ones. Moreover, from the microscopic images, the presence of some stretched shape macrophages is visible in the presence of PolyMem WIC silver (Figure 4H).

**FIGURE 5:**
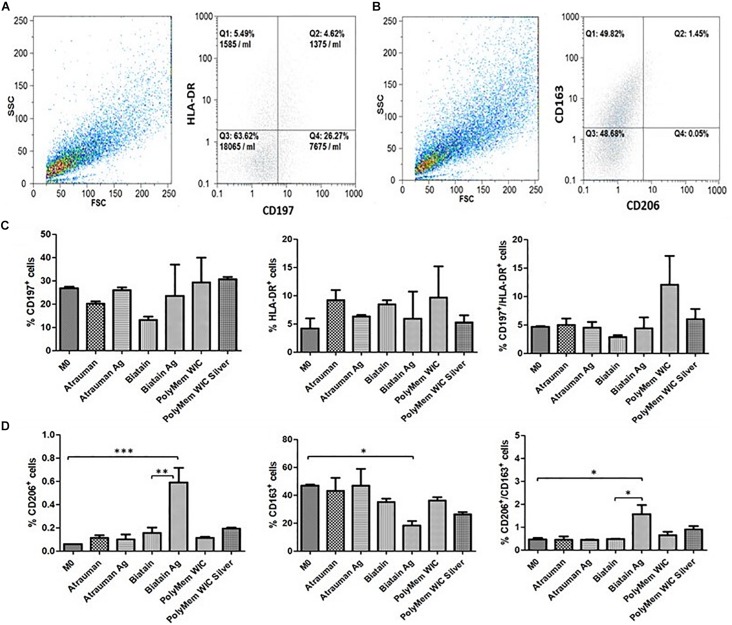
Surface markers for phenotypical characterization of macrophage populations. **(A,B)** Representative dot plots from Flow cytometry of M0 macrophages; **(C)** Percentage of single positive CD197 cells, single positive HLA-DR and double positive CD197^+^/HLA-DR. **(D)** Percentage of single positive CD206 cells, single positive CD163 and double positive CD206^+^/CD163^+^. *p* values (^∗^*p* ≤ 0.05, ^∗∗^*p* ≤ 0.01, and ^∗∗∗^*p* ≤ 0.001) were determined for M0 vs. all materials and unloaded vs respective silver containing material of three independent experiments. Error bars represent standard deviation.

**FIGURE 6:**
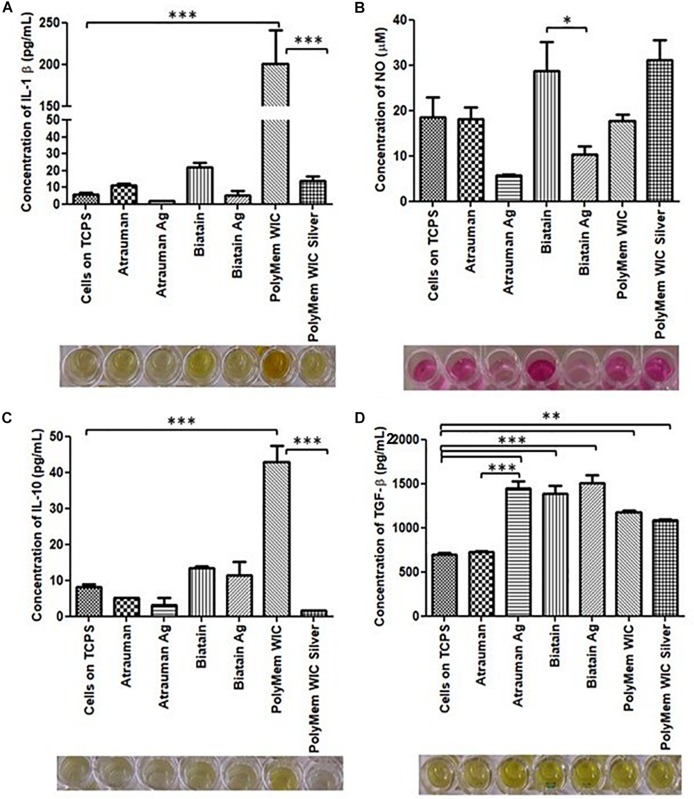
Concentration of produced pro-inflammatory and anti-inflammatory signals by macrophages in the presence of different commercially available materials for 24 h. **(A)** concentration of IL-1β; **(B)** concentration of nitric oxide (NO); **(C)** concentration of IL-10; **(D)** concentration of TGF-β. *p* values (^∗^*p* ≤ 0.05, ^∗∗^*p* ≤ 0.01, and ^∗∗∗^*p* ≤ 0.001) were determined for cells on TCPS (M0 macrophages) vs. all materials and untreated vs. respective silver containing dressing (*n* = 3). Error bars represent standard deviation.

No noticeable impact on macrophage immunomodulation was observed for macrophages in the presence of Silver-free Atrauman since the markers expressed on the membrane and levels of signals production were very similar to the M0 macrophages (Figures 5C,D, 6). After contact with silver-containing Atrauman, the THP-1 derived macrophages decreased slightly NO production and increased significantly the secretion of the anti-inflammatory TGF-β cytokine (Figures 6B,D). Both silver-free Biatain Alginate and Biatain Alginate Ag raised the levels of TGF-β (Figure 6D). An augmented expression of HLA-DR which is a M1-associated marker was detected in macrophages previously exposed to Biatain Alginate alone (Figure 5C). Interestingly, Biatain Alginate alone also induced the production of pro-inflammatory molecules: slightly higher IL-1β concentration and significantly more nitric oxide concentration in the supernatant (Figures 6A,B). However, macrophages in the presence of Biatain Alginate Ag produced reduced NO levels and a significant increase of single positive CD206^+^ cells and double positive CD163^+^/CD206^+^ on the membrane of macrophages was determined (Figures 5D, 6B). It is well observed on Figure 5B, that the previously mentioned markers are less detected in the correspondent quadrants of the plot for a representative sample of M0 macrophages population. The PolyMem dressing induced a slight increase of populations that express HLA-DR and double CD197/HLA-DR markers, that are considered pro-inflammatory receptors (Figures 5A,D). A significant increase in the production of IL-1β, IL-10, and TGF- β was observed additionally (Figures 6A,C,D). In comparison to the respective silver-free foam, the macrophages in contact with PolyMem WIC Silver maintain the induction of high TGF-β production but typical cell surface markers of M1 and pro-inflammatory cytokines are not detected anymore (Figures 5C, 6D).

**FIGURE 7:**
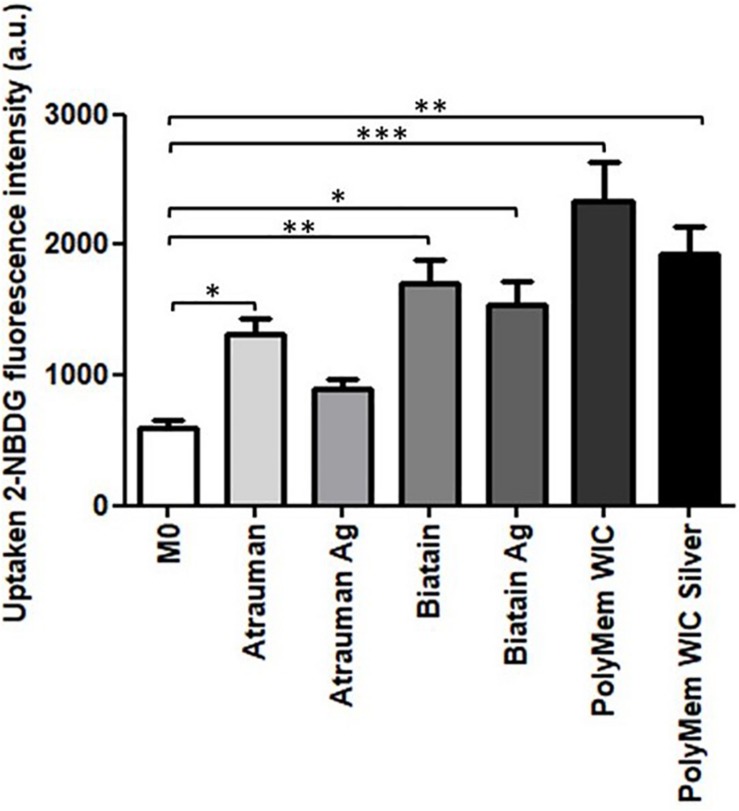
Glucose consumption by monocyte-derived macrophages after exposure to the materials during 24 h. 2-NBDG, fluorescent D-glucose analog. Error bars represent standard deviation. Statistical significance determination is represented as ^∗^*p* ≤ 0.05, ^∗∗^*p* ≤ 0.01, and ^∗∗∗^*p* ≤ 0.001 for cells on TCPS (M0 macrophages) vs. all materials, and untreated vs. respective silver containing dressing of three independent experiments.

The glucose uptake by macrophages exposed to unloaded versus silver containing dressings increased significantly in all conditions compared to M0 resting state macrophages, with exception of Atrauman Ag. Still it is clear that macrophages consume slightly less glucose in the presence of the silver containing materials (Figure 7). The most significant increase of glucose consumption occurred to macrophages that were previously exposed to PolyMem WIC (Figure 7).

## Discussion

Microbial contamination of wounds is a significant contributor for the delay of wound healing (Leaper et al., 2015). The usage of antimicrobial wound dressings has been increased in hospitals as a common practice for the prevention of infections or the reduction of bacterial burden in the wound environment. Incorporation of silver as an antimicrobial agent that is not a conventional systemic antibiotic treatment in such matrixes is one of the most common strategies. In the last years many clinical cases were reported, in which currently-used antibiotics failed in their bactericidal or bacteriostatic effect, due to the evolution and propagation of multidrug-resistant species (Blair et al., 2015).

Out of the three antimicrobial wound dressings selected for this study, Biatain Alginate Ag decreased the number of Gram-positive (*S. aureus* and *S. epidermidis*) and Gram-negative (*E. coli* and *P. aeruginosa*) bacteria significantly in the first 24 h. All materials tested achieve the antibacterial inhibition by releasing silver ions, that occurs optimally in contact with a moist or liquid environment, such as the exudate of wounds. However, these ions have a different origin for each material, and the polymeric component and the amount of silver is also different. In a short period of time, the Ag^+^ generated from the inorganic salt used in Biatain Alginate Ag, diffuses quicker than the one originated by the metallic silver incorporated into Atrauman Ag and the silver ions and particles that are formed from the nanocrystalline silver of PolyMem WIC Silver. This is probably because among all dressings Biatain Alginate Ag is the material with the highest silver amount per cm^2^ (0.95 mg/cm^2^) (Coloplast, 2015). However, in a direct contact test, it also provoked a cytotoxic outcome on fibroblasts and monocyte-derived macrophages, reducing the viability of about 50%. Nevertheless, this material is clearly stimulating the polarization of M0 macrophages to the M2-state since an increase of expression of membrane M2 markers CD163^+^/CD206^+^ and production of TGF-β was verified and a decrease of anti-inflammatory molecules induced by Biatain Alginate alone was also confirmed. Biatain Alginate consists of 85% alginate and 15% carboxymethylcellulose. Although, the information about the origin of the alginate is not openly available, it was previously reported that alginate isolated from *Sargassum vulgare* has pro-inflammatory activity (Lins et al., 2013). Hence, the M1 characteristics observed on macrophages in the presence of Biatain might be a consequence of the contact with alginate. The combination of the matrix properties with the ionic silver complex salt has a clear action in initiating the immunomodulation of macrophages toward the M2-state, possibly to M2a or M2c which are both pro-angiogenic subtypes that show similar characteristics (Novak and Koh, 2013; Hesketh et al., 2017). Therefore, with a rapid killing of microorganisms and shifting macrophages to a tissue-healing population, Biatain Alginate Ag seems promising for regressing the inflammatory perpetual state on chronic wounds which would impulse a proper cutaneous closure.

Atrauman and Atrauman Ag showed excellent cytocompatibility features on the cell lines used in this work. One specific observation was that Atrauman itself seems to be ineffective in inducing any alteration to the macrophage’s phenotype for a period of 24 h. This can be an advantage in clinical situations that no interference with the natural wound healing is demanded, such as acute wounds that eventually recover naturally and a dressing is just necessary to protect the wound from external factors. The presence of metallic silver influenced the production of TGF-β to a significant extent, which shows that silver is in fact stimulating the macrophages to secrete this anti-inflammatory signal. In this case, it seems that the effect comes from the Ag^+^ ions released by the matrix that have been reported to have anti-inflammatory properties (Hermans, 2006; Leaper, 2006; Wilkinson et al., 2011; Lansdown, 2014; Rosique et al., 2015). As observed in disk diffusion, no antibacterial effect was detected against *S. aureus* and *P. aeruginosa* (Figure 1). These results are not in accordance with previous studies performed by the material developers, that observed bacterial reductions of all strains within 24 h. However, their antibacterial evaluation method was entirely different from the ones applied in the present study. The EUCAST guidelines followed here were performed to have uniformity in all tests with the different wound dressings, whereas in the other company’s study the standard method 2180 of the American Society for Testing Materials was used, in which the melted agar was inoculated with the different bacterial strains and then dispersed onto the dressing to be examined (Ziegler et al., 2006). In the latter method, bacteria are “forced” to grow on the silver dressing creating an enclosed environment in which the thin agar inoculated layer is in closer contact with the wound dressing. This might be an explanation for the different results, because in both methods applied in our study bacteria are still growing on an optimal surface (MHB agar) or in a nutritious medium (MHB broth), probably avoiding the antibacterial effect from the Atrauman Ag dressing *in vitro*. It is also important to notice that metallic silver is relatively inert and releases biocidal Ag^+^ ions when interacting with moisture and wound fluid on skin, which have different constituents than the ones existing on MHB (Qin, 2009). This can be a factor that influenced the release of silver ions.

The polarization of macrophages involves plasticity in a continuous spectrum. Hence, in the case of PolyMem alone, macrophages might be shifting from a M1- to M2-state or be part of a mixed activation of M1/M2 phenotypes population (Bosurgi et al., 2011; Dalmas et al., 2011). This observation is not in accordance with the product description on the company website, in which it is strongly defined that PolyMem WIC (that contains glycerol and poloxamer 188) is an anti-inflammatory dressing. As published by Szél et al. (2015) glycerol is a simple polyol with anti-inflammatory properties Additionally, the anti-inflammatory effect induced by the non-ionic block copolymer surfactant Poloxamer 188 has also been confirmed (Harting et al., 2008). Hence it becomes clear that the increase on the production of the anti-inflammatory cytokines IL-10 and TGF-β is due to stimulation by glycerol and poloxamer 188, both present in this product. The reason concerning the increase on IL-1β secretion and expression of typical pro-inflammatory membrane markers is not yet fully understood and it needs to be further investigated. It cannot be ruled out to be an artifact of the experimental design. Nonetheless, a significant drop on IL-1β production was observed when the macrophages were in the presence of PolyMem WIC with the nanocrystalline silver particles, proving once again the anti-inflammatory impact induced by silver ions and particles. This antimicrobial dressing showed a great antibacterial effect against the most common strains found in infected wounds exclusively in liquid medium. PolyMem WIC Silver was studied previously against *S. aureus* and *P. aeruginosa* and the same lack of antibacterial effect was observed on the agar disk diffusion test (Yunoki et al., 2015). Thus, the release of clusters of reactive small particles and silver ions from the nanocrystalline silver seems to be very reduced on agar. Moreover, Boonkaew et al. (2014) observed a zone of inhibition against *Staphylococcus aureus* if the material is pre-activated in 0.85% saline solution by inducing its swelling, before it is placed on the agar plate. Hence, it becomes clear that the fluid taken up by this polyurethane foam plays a major role in unleashing the antimicrobial power of nanocrystalline silver. Among all the dressings tested, it was noted that PolyMem and PolyMem WIC Silver were highly absorbent, entrapping a part of the liquid medium in the foam material. This was also observed by Burd et al. (2007) in which this absorbency capacity was associated with the very low release of silver ions measurement still in the presence of a significant antibacterial effect. This result lead the authors to conclude that PolyMem WIC silver dressing induces its antibacterial effect mainly by “soaking” the pathogenic bacteria and its contaminants into the foam structure and induce its “killing” internally. In addition, the same authors stated that this could be a benefit, because it will decrease the possibility for toxicity on the healthy wound cells (Burd et al., 2007). However, this dressing brought a toxic effect to the 2D cell culture of fibroblasts, keratinocytes and monocyte-derived macrophages.

All silver-containing dressings tested induced a significant increase of TGF-β secretion, which has profibrotic activities and is associated to M2a and M2c subsets of M2 macrophages (Lu et al., 2013). Profibrotic action means that it promotes fibrosis which involves the overgrowth of an organ or tissue during a reparative process, by deposition of ECM components such as collagen (Wynn, 2008). Also, it was detected that macrophages consume slightly less glucose in the presence of the silver containing materials, demonstrating that these macrophages are polarizing on a continuous spectrum toward M2-macrophages (Figure 7) since an increased expression of glucose transporters (mainly GLUT1) on the membrane and higher rates of consumption of glucose has been associated to pro-inflammatory M1 macrophages. This is due to their metabolic adaptation in which they perform fermentation of glucose to obtain a rapid production of energy (Freemerman et al., 2014).

Although this study gives significant indications about the response of macrophages to different clinically applied biomaterials, the macrophages used were in a resting state (M0 macrophages), hence in future studies it is necessary to investigate the immunomodulatory effects of these same dressings on macrophages polarized to the M1 pro-inflammatory state due to the fact that in the wound environment this macrophage phenotype is predominant. It would be interesting to explore the same features with monocytes isolated from donors’ blood, to have an *in vitro* set closer to human conditions. However, the main advantage of using a cell line such as THP-1 human monocytes is that the origin of cells is consistent in every assay, meaning less variability in the results obtained. Hence, these cells are anyway an effective tool to give insights of immunomodulation owed by biomaterial-macrophages interactions.

In addition, there was a decrease of viability of monocyte-derived macrophages in the presence of Biatain Alginate Ag, PolyMem WIC and PolyMem WIC Silver after 24 h. Toxicity of PolyMem Silver and Biatain Alginate Ag has been reported before, on *in vitro* indirect contact studies on diabetic fibroblasts, in which the authors registered a decrease in cell viability of more than 50% (Zou et al., 2013). Moreover, cytotoxicity of PolyMem Ag has been described in Yunoki et al. (2015) research article, in which the authors attribute the negative effect on mammalian cells to the uptake of silver clusters of large size by endocytosis. This may have influenced the results of the measured concentrations of pro-inflammatory and anti-inflammatory molecules, and the uptake of glucose. Nevertheless, in comparison with M0 macrophages, secretion of IL-1β, IL-10, and TGF-β increased significantly in macrophages in contact with PolyMem WIC. Also, for Biatain Alginate Ag and PolyMem WIC Silver the production of TGF-β was significantly higher than the M0 state, and the glucose uptake was increased in cells at the presence of the three wound dressings. Hence, although a smaller number of cells were attached to the bottom of the wells by the endpoint of the assay, higher concentrations of secreted cytokines in the supernatant and more glucose uptake were still detected.

## Conclusion

One main finding of this work is that macrophages start to change their metabolism and phenotype in response to wound dressings of different composition in a short 1-day period. Moreover, silver has shown to possess anti-inflammatory properties. The other major final remark from this research work is, that it is clear that even with commercially available and clinically applied products the balance between antibacterial activity versus cytotoxicity *in vitro*, and stimulation of an optimal wound environment toward cutaneous-healing remains a significant challenge.

## Data Availability Statement

All datasets generated for this study are included in the article/supplementary material.

## Author Contributions

PV planned and performed all the experiments, conducted the treatment and interpretation of data, and contributed substantially to the writing of this article. LM helped in experimental work related to microbiological assays. SS and RV contributed to the revision of the manuscript. GC and JS contributed to the planning of experiments, interpretation of data, and manuscript writing and revision.

## Conflict of Interest

The authors declare that the research was conducted in the absence of any commercial or financial relationships that could be construed as a potential conflict of interest.
